# Complete pathological response following neoadjuvant FOLFOX chemotherapy in BRCA2-mutant locally advanced rectal cancer: a case report

**DOI:** 10.1186/s12885-018-5182-z

**Published:** 2018-12-14

**Authors:** Zhenyu Lin, Junli Liu, Li Peng, Dejun Zhang, Ming Jin, Jing Wang, Jun Xue, Hongli Liu, Tao Zhang

**Affiliations:** 1 Cancer Center, Union Hospital, Tongji Medical College, Huazhong University of Science and Technology, Wuhan, 430022 China; 2 Department of Pathology, Tongji Medical College, Union Hospital, Huazhong University of Science and Technology, Wuhan, 430022 China

**Keywords:** Rectal cancer, Neoadjuvant chemotherapy, BRCA; mutation load

## Abstract

**Background:**

Patients with locally advanced rectal cancer (LARC) achieving a pathological complete response (pCR) to neoadjuvant treatment usually have a good prognosis, but only accounted for less than 20%.

**Case presentation:**

We report a case of a 25-year-old male with LARC treated with neoadjuvant FOLFOX chemotherapy, and experienced a pCR. The next-generation sequencing analysis revealed the presence of breast cancer gene 2 (BRCA2) somatic mutation and an increased somatic mutational load without microsatellite instability (MSI). To our knowledge, this is the first report of BRCA2 mutant LARC that demonstrated significant benefit from FOLFOX neoadjuvant treatment.

**Conclusions:**

This case indicated an association of BRCA2 mutation with high mutation loads and an excellent response of oxaliplatin-based chemotherapy regimen for LARC. Our findings encourage further studies to analyze BRCA mutations in patients with LARC, especially for those patients unable or unwilling to receive radiotherapy.

## Background

With the implementation of total mesorectal excision (TME) surgery and neoadjuvant concurrent chemoradiotherapy, local recurrence rates of LARC have declined from 30 to 50% to less than 10% [1]. However, no clear benefit of radiotherapy in terms of overall survival was demonstrated. Moreover, it increases surgical morbidity, consequently delaying the administration of subsequently adjuvant chemotherapy, impacts anorectal, sexual and urinary functions and is related with an increased risk of secondary malignancies, especially in those young patients [2].

Pathologic complete response is defined as no residual viable invasive adenocarcinoma cells within the parenchyma in a neoplastic tissue specimen. For several different cancer types, pCR is associated with lower incidence of local recurrences, distant metastases, and better overall survival rates [3]. The concept of neoadjuvant chemotherapy has recently emerged due to the introduction of more effective chemotherapy treatment. However, a low pCR rate is still inevitable in the current practice of LARC. Moreover, there is no biological marker that can reliably predict pathological response during the course of treatment. We herein present a case of young LARC patient who received only neoadjuvant chemotherapy with FOLFOX and presented a pCR. Next generation sequencing (NGS) analysis exhibited presence of the BRCA2 somatic mutation and high mutation loads, which might be associated with its excellent response. To the best of our knowledge, this is the first case report of BRCA2-mutuant locally advanced rectal cancer that experienced a complete response from neoadjuvant chemotherapy of FOLFOX treatment.

## Case presentation

**Fig. 1:**
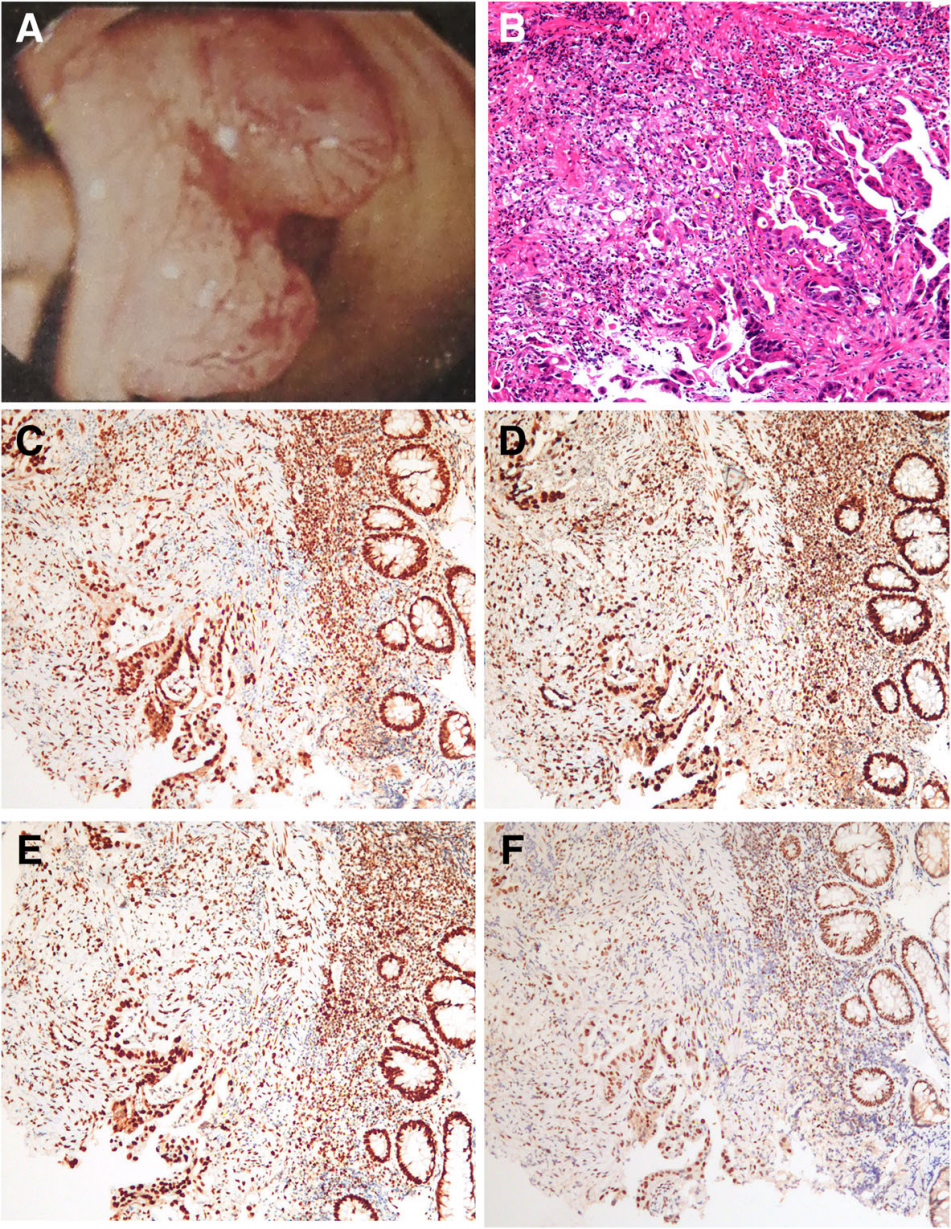
Endoscopic and pathological features of rectal cancer. (**a**): Colonoscopy showed a circumferential mass at the middle rectum; (**b**): Photomicrographs showing the cytomorphological findings of endoscopic biopsy specimens. Hematoxylin and eosin section shows tumor cells have round-to-ovoid nuclei with dense chromatin, and moderate amount of eosinophilic cytoplasm (original magnification × 100). (**c**-**f**): Photomicrographs showing positive staining of DNA mismatch repair proteins MLH1 (**c**), MSH2 (**d**), MSH6 (**e**) and PMS2 (**f**) in biopsy specimens. (original magnification × 100)

**Fig. 2:**
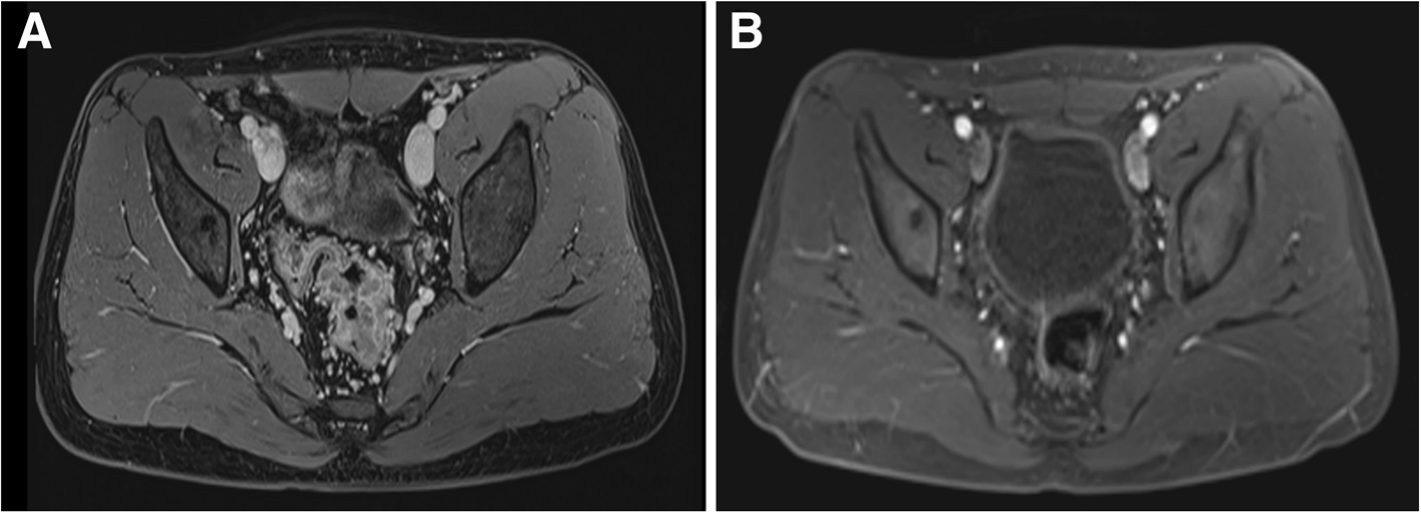
Magnetic resonance imagines (MRI) of the pelvis (**a**): Sagittal MRI of the pelvis showed rectal mass with involvement of perirectal fat and enlarged lymph nodes. **b**: Following 4 cycles of FOLFOX neoadjuvant chemotherapy, the follow up MRI reveals significant tumor regression, indicating response to treatment

**Fig. 3:**
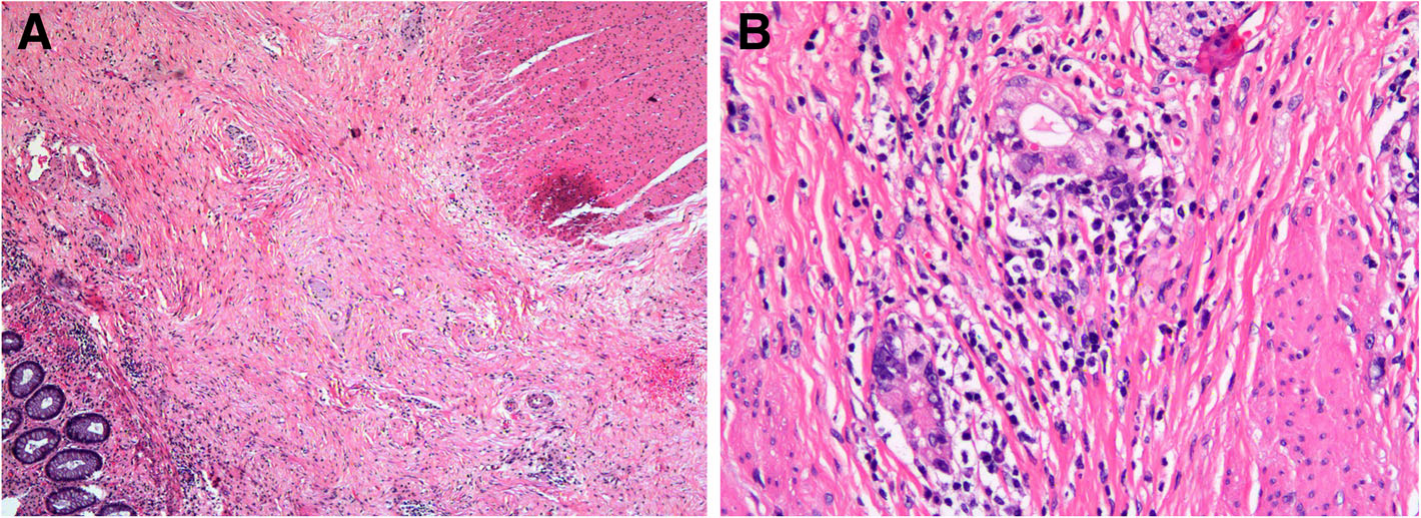
Photomicrographs showing the cytomorphological findings of rectal specimens following surgical resection. **a**: low power view of section from resected rectum showing fibrosis of the previous tumor bed and a repair reaction (original magnification × 40). **b**: Higher magnification from the fibrotic area showing fibroblasts laying down collagen and a scattering of inflammatory cells (original magnification × 200)

A 25-year-old male presented to our hospital with a one-month history of bloody stool. He denied any tobacco or alcohol use. His serum level of carcinoembryonic antigen (CEA) was 21.0 ng/mL (normal < 2.5).The colonoscopy examination revealed a circumferential rectal lesion at 7 cm from the anal verge (Fig. 1a). An endoscopic biopsy confirmed the diagnosis of poor differentiated adenocarcinoma (Fig. 1b). Immunohistochemical staining for mismatch repair proteins MSH6 (BD Transduction Laboratory; clone 44, 1:1000 dilution), MSH2 (Calbiochem, Oncogene Sciences; clone FE11, 1:100 dilution), MLH1 (BD Biosciences Pharmingen; clone G168–15, 1:100dilution) and PMS2 (BD Pharmingen; clone A16–4, 1:500 dilution) was performed as described previously [4]. And the results showed proficient mismatch repair (pMMR) for this patient (Fig. 1c-f). Magnetic resonance imaging (MRI) of the pelvis revealed heterogeneously enhancing irregular circumferential mural wall thickness involving the mesorectal fat measuring 5 cm in its length with enlarged perirectal lymph nodes (Fig. 2a). The computed tomography findings of the chest were negative. Based on the above findings, he was diagnosed with rectal cancer (cT3N2M0). The patient was originally planned to receive standard preoperative 5-FU-based CRT. However, he refused any radiotherapy concerning the increased risk of radiation-induced infertility and received 4 cycles of neoadjuvant chemotherapy with mFOLFOX6 regimen that consisted of oxaliplatin 85 mg/m^2^ intravenously, leucovorin 400 mg/m^2^ intravenously followed by fluorouracil 400 mg/m^2^ intravenously and fluorouracil 2.4 g/m^2^ by 48 h continuous intravenous infusion. The patient experienced only grade I gastrointestinal and hematological toxicities, after which he underwent re-staging with CT or MRI scans of the chest, abdomen, and pelvis. Re-examination of pelvic MRI 4 weeks after diagnosis revealed evidence of tumor regression (Fig. 2b). Consequently, the patient received laparoscopic TME for rectal cancer, and histological examination of the surgical specimen revealed chronic ulcerative lesions but no evidence of residual adenocarcinoma cells (Fig. 3), consistent with the concept of a complete response according to the AJCC TRG system. All 13 lymph nodes resected were negative (ypN0). Another 8 cycles of mFOLFOX6 adjuvant therapy was given following surgery. His CT scans showed no evidence of recurrence3 and 6 months after operation, and CEA is normal. No evident recurrence has been found in the patient for 12 months.

Given the significant tumor response, we next performed next-generation sequencing (NGS) using DNA from the endoscopic specimen as described [5]. DNA sample was extracted from the endoscopic specimen and peripheral blood samples (DNeasy Blood and Tissue Kit, QIAGEN, Hilden, Germany). After quality control, DNA sequencing libraries were prepared according to the protocols recommended in the Illumina TruSeq DNA Library Preparation Kit (Illumina, San Diego, CA, USA). Libraries were hybridized to custom-designed biotinylated oligonucleotide probes (Roche NimbleGen, Madison, WI, USA) covering ~ 1.1 Mbp of sequence. DNA sequencing was carried out with the HiSeq CN500 Sequencing System (Illumina, San Diego, CA, USA). After removing the terminal adaptor sequences and low-quality data, the reads were mapped to the reference human genome. GATK (https://software.broadinstitute.org/gatk, The Genome Analysis Toolkit) and MuTect were used to call small insertions and deletions (indels) and single nucleotide variants in the somatic DNA by filtering peripheral blood sequencing data. Out of our expectation, a total of 159 somatic mutations were identified with *TP53* p.R213* as the mutation with the highest allele frequency (53.3%). Moreover, *KRAS* p.G12D mutation was also detected at the allele frequency of 51.4%. Intriguingly, presence of *BRCA2* somatic mutation (c.2231C > A p.S744*) was observed. The number of somatic mutations detected on NGS (interrogating 1.2 mb of the genome) is quantified and that value extrapolated to the whole exom. TMB was measured in mutations per megabase (Mb) and classified into high (20 mut/Mb), intermediate (6–19 mut/Mb) and low (< 6 mut/Mb). Consequently, the tumor mutation burden of our patient was described as high (22 mut/Mb).

## Discussion and conclusions

A pathological complete response to neoadjuvant treatment in LARC patients usually indicates a good prognosis, but the proportion is quite small. In the era of TME surgery, radiotherapy has not shown a conclusive benefit of overall survival but is associated with safety concern. Given that the local recurrence rate for TME alone is less than 10%, the benefit of neoadjuvant radiotherapy should be carefully weighed against potential negative effects [6]. Therefore, strategies to improve tumor response and alleviate side effects can alter the present treatment algorithm by maximizing the proportion of patients eligible for less approach.

The essential goal of neoadjuvant treatment for LARC is to increase the likelihood of R0 resection and to eliminate micrometastatic lesion. The idea of neoadjuvant chemotherapy without radiation has not been assessed in resectable rectal cancer until recently because of the risk of disease progression and overtreating patients. Neoadjuvant chemotherapy without radiotherapy has been investigated for the treatment of LARC in several small trials. And promising results were observed for lower toxicity than observed in patients who received chemoradiotherapy, indicating it as a further option for patients unable or unwilling to receive radiotherapy [7]. For example, Deng et al. showed perioperative mFOLFOX6 alone had a similar downstaging rate as fluorouracil-radiotherapy, with less toxicity and fewer postoperative complications. However, the incidence of pCR rate was only 6.6% [8]. Consequently, the identification of a good biological marker for predicting pCR to neoadjuvant chemotherapy is urgently needed.

Chemotherapy regimen containing oxaliplatin is commonly used in the management of rectal cancer. Approximately 50% of patients benefit from treatment with oxaliplatin [9]. Mutations of the BRCA1 (breast cancer gene 1) and BRCA2 (breast cancer gene 2) genes are considered biomarkers of genomic instability and DNA damage repair deficiency and used as predictive biomarkers of response to platinum-based agents and PARP inhibitors [10]. The BRCA1 and BRCA2 genes confer increased susceptibility to ovarian and breast cancers. However, the risk of rectal cancers patients associated with BRCA mutations remains controversial [11]. And the incidence of BRCA mutations in rectal cancer has not been previously reported. Accumulating evidence suggests significant effect of cisplatin or oxaliplatin in BRCA associated malignancies. The survival of patients with BRCA-associated hereditary ovarian cancers is longer than that of nonhereditary cancer patients [12]. However, Kotsopoulos et al. reported that BRCA mutations only reflected a higher initial sensitivity of BRCA carriers to chemotherapy, but not predicted long-term survival [13]. In breast cancer, neoadjuvant chemotherapy resulted in pathological complete response of 83% in 12 cases of BRCA1 carrier patients [14]. And pathological complete responses to oxaliplatin or cisplatin have also been reported in pancreatic adenocarcinoma with germline BRCA2 mutations [15]. However, case of somatic BRCA mutant rectal cancer has not been reported in the literature before. Therefore, we felt that it was critical to describe this case of a significant clinical response to neoajuvant oxaliplatin-based therapy in a LARC patient who carried a BRCA2 mutation. During the review process of the article, Aixa E. et al. also reported a case of a young man with LARC and a germline BRCA1 pathogenic variant. The patient was treated with neoadjuvant FOLFOX chemotherapy and radiotherapy, and also experienced a pCR [16]. This is a further confirmation of our study about the association of BRCA mutation with an excellent response of oxaliplatin-based chemotherapy regimen in rectal cancer. Although the BRCA2 mutation may present in only a minority of rectal cancers, the excellent response of these group to oxaliplatin-based chemotherapy regimens indicates the BRCA status might provide an attractive predictive marker for the efficiency of oxaliplatin in the treatment of LARC.

Immune checkpoint inhibitors have emerged as a potent new class of anticancer therapy. And mismatch repair-deficiency or MSI has recently been demonstrated to predict clinical benefit to immune checkpoint inhibition therapy in metastatic colorectal cancer. However, patients without MSI may also benefit from Immune checkpoint inhibition. A recent NGS study found that increased mutational load was significantly correlated with MSI yet colorectal tumors with the highest mutational burden that were distinct from MSI tumors all harbored POLE mutations [17]. The characterization of mutational load in colorectal cancer (CRC) may serve as a better marker than MSI status in determining a hypermutant profile that could predict clinical benefit from immunotherapy [18]. Another interesting point in our patient is high mutational burdens without MSI. Although we have notdemonstrated a direct link between high mutational burdens and BRCA somatic mutation, our observations are consistent with those recently reported for triple-negative breast cancers and serous ovarian cancer, where higher neoantigen loads observed in BRCA1/2-mutated tumors compared to DNA repair–proficient tumors [19, 20]. Considering the higher mutational load and unique mutational signature, these features provide a rationale for exploring the utility of checkpoint inhibitors in BRCA2-mutated rectal tumors. Due to the low MSI rate (15%) in colorectal cancers, BRCA mutation testing might be considered when the diagnosis of rectal cancer is found.

In conclusion, the current study demonstrated a rare occurrence of complete pathological response in a BRCA2-mutuant locally advanced rectal cancer patient treated with neoadjuvant FOLFOX chemotherapy. This case suggests an association of BRCA2 mutation with high mutation loads and an excellent response of oxaliplatin-based chemotherapy regimen for LARC. Our findings encourage further studies to analyze BRCA mutations in patients with LARC, especially for those patients unable or unwilling to receive radiotherapy.

## Abbreviations

BRCA2: breast cancer gene 2
CEA: carcinoembryonic antigen
CRC: colorectal cancer
LARC: locally advanced rectal cancer
MRI: magnetic resonance imaging
MSI: microsatellite instability
NGS: next generation sequencing
pCR: pathological complete response
TME: total mesorectal excision
